# No evidence of SARS-CoV-2 infection in *Rousettus aegyptiacus* bat in Egypt

**DOI:** 10.1080/23144599.2021.1991135

**Published:** 2021-10-28

**Authors:** Omar Sayed Saeed, Ayman Hany El-Deeb, Hussein Aly Hussein Ahmed

**Affiliations:** Department of Virology, Faculty of Veterinary Medicine, Cairo University, Giza, Egypt

**Keywords:** *Rousettus aegyptiacus* bat, SARS-CoV-2, spillover

## Abstract

Bats are considered ideal reservoirs for zoonotic viruses with emerging capabilities over the past two decades and spotted evidence points out that they may play a role as a reservoir host for SARS-CoV-2. To investigate the possible role of bats as part of SARS-CoV-2 anthropozoonotic spill-over infections in Egypt, a total of 800 samples obtained from 200 Egyptian fruit bats (*Rousettus aegyptiacus*) were tested for SARS-CoV-2 using quantitative RT-PCR assay (RT-qPCR). RT-qPCR analysis of RNA extracted from bat tissues showed no positive results for SARS-CoV-2 nucleic acid. These findings suggest that during the study period, the *Rousettus aegyptiacus* bat was not a reservoir or amplifying host for SARS-CoV-2 infection in Egypt. The lack of SARS-CoV-2 nucleic acid in Egyptian fruit bats is thought to make a significant contribution to SARS-CoV-2 epidemiology.

## 1. Introduction

The coronavirus disease 2019 (COVID-19) pandemic was caused by a previously unknown coronavirus, called severe acute respiratory syndrome coronavirus 2 (SARS-CoV-2) **[**1**]**. Data from the World Health Organization show that as of the 1^st^ of Oct 2021, about 233 million individuals have reported being affected by the virus including more than 4.5 million who have surrendered to this attack globally **[**2**]**.

In the past 20 years, two β-coronaviruses emerged: severe acute respiratory syndrome coronavirus (SARS-CoV) in China 2002 and Middle East respiratory syndrome coronavirus (MERS-CoV) in Saudi Arabia 2012 **[**3,4**]**. SARS and MERS coronaviruses are thought to have originated in bats but have further adapted to animal hosts such as palm civets and dromedary camels, respectively, from which sustained spillover infections have occurred **[**5**]**. SARS-related-CoVs (SARSr-CoVs) were detected in horseshoe bats in Hong Kong, similar to the findings for SARS-CoV and MERS-CoV **[**6**]**. Hence, bats are considered to be exceptional mammals that provide a large genomic pool for the emergence of novel human coronavirus **[**7**]**. The coronavirus disease 2019 (COVID-19) was first reported in Egypt in March 2020, with a dramatic rise in the number of illnesses since then. In addition to the health and economic challenges COVID-19 posed, there are also several challenges identified at different stages of the pandemic, with several key scientific queries remaining unelaborated. One of them is the obscure origin of SARS-CoV-2 and its transmission event from animal to human **[**8**]**. The *Rousettus aegyptiacus* bat is the most abundant fruit bat species in Egypt, dwelling in abandoned structures and fruit gardens close to humans, indicating the possibility of zoonotic virus transmission **[**9**]**. Although the COVID-19 pandemic may have begun with a bat-to-human transmission event, the exact reservoir of SARS-CoV-2 remains obscure **[**10**]**. In the present study, we aimed to investigate the potential presence of SARS-CoV-2 in *Rousettus aegyptiacus* bat from different governorates in Egypt.

## 2. Materials and methods

### 2.1. Ethical statement

All the bat captures were conducted under the approval of the Institutional Animal Care and Use Committee, Faculty of Veterinary Medicine, Cairo University, number (Vet CU28/04/2021/274).

### 2.2. Bat sampling

Two hundred fruit bats were caught and sampled at different roosting sites, caves and mines across different Egyptian governorates (Table 1). The bats were captured using nylon mesh mist nets; they were later morphologically identified according to standard methods developed by Dietz et al. **[**11**]** into the family *Pteropodidae*; genus *Rousettus*. Bats were held; wingspan, forearm length and tail were measured by mechanical caliper. Moreover, the ear of Egyptian fruit bat is simple without tragus or antitragus and the second finger is obviously clawed.

**Table 1.** Bat samples collected in the Egyptian governorates in 2020–2021 tested for SARS- CoV-2

No. of bats	No. of tested organs	Sampling site	Collection peroid
35	140	South Sinai	March-May 2020
40	160	Asyut	
40	160	Giza	Jan – April 2020
45	180	Qena	
20	80	Sohag	Sep 2020-April 2021
20	80	Minya	

Eight hundred samples including liver, spleen, intestine and lung tissues were collected from all individuals (four organs from each individual 4×200).

### 2.3. RNA extraction and molecular characterization

All organs were inactivated in RNAlater™ Stabilization Solution (Thermo fisher scientific, #AM7021), stored at −80°C till testing. Viral RNA was extracted directly from tissues from all individuals using RNeasy Mini Extraction kit (Qiagen, #74,104) following the manufacturer’s instructions. Extracted RNA was eluted in 50 µL of RNase-free water and stored at −80°C prior to use. Screening of SARS-CoV-2-related RNA was performed by RT-qPCR targeting RdRp, the highly conserved gene among beta coronaviruses as described in the work of Corman et al. [12]. RT-qPCR was conducted using Verso 1-step RT-qPCR Kit (Thermo Fischer Scientific, #AB4100A) with positive control for the nucleic acid extraction process, the bat endogenous gene GAPDH as internal control and positive RNA of characterized SARS-CoV-2 human sample (external control) that were amplified in parallel. Water instead of nucleic acid was used as a negative control. All samples were tested in batches with positive and negative controls included in each run.

A 25 μL reaction contained 5 μL of extracted RNA, 12.5 μL 2X 1-Step qPCR Mix provided with 1.25 μL RT Enhancer, 0.25 μL of verso enzyme mix from the kit (Thermo Fischer Scientific, #AB4100A), 1 µL per each forward primer and reverse primer and H2O up to 25 µL. All primer oligonucleotides were synthesized and provided by Bio Basic (Toronto, Canada). All samples were run on a Step-one plus real-time PCR thermal cycler using the following thermal cycling conditions: 55°C for 10 minutes for RT, followed by 95°C for 3 min and then 45 cycles of 95°C for 15 seconds and 58°C for 30 seconds.

## 3. Results and discussion

Although rapid detection methods for the detection of SARS-coV-2 antigen are now broadly used, real-time quantitative reverse transcription PCR (RT-qPCR) due to its sensitivity and specificity will still be the gold standard for the diagnosis of COVID-19 cases [13]. In addition, RT-qPCR assay is the recommended method for recognizing and laboratory confirmation of COVID-19 cases because these methods have been evaluated by the World Health Organization (WHO) for quality and safety [14]. The protocol for the first RT-qPCR test, focusing on RNA-dependent RNA polymerase (RdRp), SARS-CoV2 envelope (E) and nucleocapsid (N), was actually proclaimed very early in late January 2020 [15]. Furthermore, cross-reactivity is a prevalent concern in serological testing, which is exacerbated by the fact that SARS-CoV-2 and SARS-CoV-1 have about 80% genetic identity and that major proteins possess structural homology with SARS-CoV-1 [16–18].

The presence of SARS-COV-2 nucleic acid in the tested organs was not demonstrated by RT-qPCR analysis of RNA extracted from harvested tissues of all captured bats. According to these findings, no SARS-COV-2 infection was found in the captured bats. This is consistent with previous findings of low-level SARS-CoV-2 replication in the same species of bats [19]. Although it is impossible to rule out the possibility that geographic sampling locations, season, and tissue targets were insufficient, or infectious phases were missed, it should be noted that beta coronaviruses and SARSr-CoVs were detected in a sample size greater than that of the present study [20]. SARS-CoV-2 and related coronaviruses were apparently obtained from a member of the *Rhinolophidae* bat family, and it is probable that this virus is limited to this bat family[21,22]. This virus may not exist in Egyptian bats due to phylogenetic variations in host and/or pathogen and thus was not discovered. To fully assess the potential role of bats in the ecology and epidemiology of SARS-CoV-2, more intensive surveillance of larger sample size, as well as conducting serological tests and viral isolation attempts, would be required.

## 4. Conclusion

There was no evidence of SARS-CoV-2 infection in *Rousettus aegyptiacus* bat in Egypt during the period of study.
